# Dose of intra‐operative opioids has no impact on recurrence or survival in primary liver cancer

**DOI:** 10.1002/cam4.4827

**Published:** 2022-05-19

**Authors:** Liuyuan Zhao, Lei Teng, Wenhui Zhang, Shiyan Lin, Xuejiao Liu, Junzhu Dai, Hongxue Shao, Xiaoshi Li, Quan Liu, Huichao Zou

**Affiliations:** ^1^ Department of Pain Medicine Harbin Medical University Cancer Hospital Harbin China; ^2^ Department of Biochemistry School of Medicine, Southern University of Science and Technology Shenzhen China

**Keywords:** hepatectomy, intro‐operative opioid, overall survival, primary liver cancer, recurrence‐free survival

## Abstract

**Background:**

Intra‐operative use of opioid analgesics might have an impact on cancer recurrence and survival after surgery. The objective of this study was to investigate the association between the intra‐operative fentanyl equivalents and survival outcomes in patients with primary liver cancer after receiving hepatectomy.

**Methods:**

This was a retrospective single‐center cohort study, and clinical data of 700 patients with primary liver cancer who underwent hepatectomy in Harbin Medical University Cancer Hospital from September 2013 to August 2018 were reviewed. After propensity matching, 376 patients were included. Patients were divided into high‐dose and low‐dose groups according to the median intra‐operative fentanyl equivalents (1.500 mg). Kaplan Meier curve and Cox proportional hazards regression model were used.

**Results:**

Results of univariable analysis showed there were no significant differences in recurrence‐free survival (RFS) (*p* = 0.136) and overall survival (OS) (*p* = 0.444) between high‐dose fentanyl equivalents and low‐dose fentanyl equivalents group. The multivariable Cox regression analysis found that the dose of intra‐operative fentanyl equivalents was not associated with RFS (HR: 1.119, 95%CI: 0.851–1.472, *p* = 0.422) or OS (HR: 0.939, 95%CI: 0.668–1.319, *p* = 0.715).

**Conclusions:**

The amounts of intra‐operative fentanyl equivalents had no impact on recurrence‐free or overall survival in patients with primary liver cancer after curative hepatectomy.

## Graphical abstract

Kaplan‐meier curves for recurrence‐free and overall survial of high‐dose and low‐dose groups. No significant difference in recurrence‐free survial (A) or overall survial (B) after surgery was noted when comparing high‐dose with low‐dose groups.

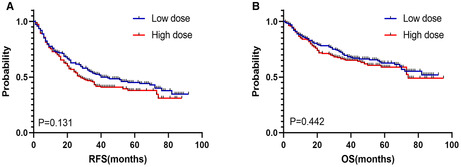

## 1 INTRODUCTION

Primary liver cancer is the sixth most common type of cancer and the fourth cause of cancer mortality worldwide.^1^ In China, nearly 400,000 people are diagnosed with liver cancer, and around 368,000 people die from it every year.^2^ Surgical treatment is the most important approach for achieving long‐term survival for primary liver cancer.^3^ However, stress during the surgery may suppress immunity which increases the risk of dissemination of tumor cells and formation of micro‐metastases.^4^, ^5^ Opioids are the mainstay of analgesics for perioperative acute pain. There are evidence that opioids suppress cellular and humoral immune function in humans.^6^ The effect of opioids on immunity may play a role in the process of cancer recurrence after surgical resection. Retrospective studies in lung,^7^ prostate,^8^ kidney,^9^ larynx,^10^ and oral cancer^10^ found that higher dose of intra‐operative opioid administration was associated with an increased risk of cancer recurrence. However, no study has assessed the effects of intro‐operative use of opioids on survival outcomes in primary liver cancer. We performed this retrospective study to investigate whether the intra‐operative fentanyl equivalents were associated with survival outcomes after hepatectomy in patients with primary liver cancer.

## 2 METHODS

### 2.1 Study design and patient selection

This was a retrospective study which was approved by the institutional review board of Harbin Medical University Cancer Hospital. Data of 700 patients with liver cancer who underwent hepatectomy from September 2013 to August 2018 were reviewed. Exclusion criteria included incomplete data, history of surgery or having other types of cancer.

### 2.2 Anesthetic management

Sulfentanil 0.4 μg/kg or fentanyl 4 μg/kg, propofol 2 mg/kg, and midazolam 0.05 mg/kg were used for anesthesia induction. Rocuronium 0.3 mg/kg or cisatracurium 0.6 mg/kg was used for muscle relax. Anesthesia was maintained with remifentanil 0.3 μg/(kg.min) combining with propofol 4 mg/(kg.h) or sevoflurane 1–3 vol%. The total amount of opioids was calculated as fentanyl as follows: 1 μg of fentanyl equal to 0.1 μg of sulfentanil, 1 μg of remifentanil and hydromorphone 10 μg.^7^

### 2.3 Variables

Data includes age, gender, body mass index (BMI), histopathological classification, tumor stage (AJCC for TNM classification), tumor size, with or without tumor thrombus, American Society of Anesthesiologists (ASA) classification, intra‐operative fentanyl equivalents, duration of operation, with or without blood perfusion, whether there were comorbidity of diabetes or hypertension. They were retrieved from the electronic medical record of Harbin Medical University Cancer Hospital.

### 2.4 Clinical outcomes

Recurrence‐free survival (RFS) and overall survival (OS) were used as primary outcomes. Recurrence‐free survival was defined as the first recurrence or death due to any causes whichever happened first. OS was defined as the time from the date of surgery to the date of death due to any cause. Patients those remaining disease free were censored in the statistical model (administrative censoring).

### 2.5 Statistical analysis

Continuous variables were presented as median and range, which were analyzed by the student *t* test. Frequency counts and percentages were defined as categorical variables. Chi‐square test or Fisher exact test was used for analysis in two categorical variables. Kaplan–Meier method was used to analyze RFS and OS. All variables were analyzed by univariable Cox model and the Log‐rank test. Cox proportional hazards regression model was used to investigate the independent predictors of RFS and OS after univariable analysis, including significant variables in univariable analysis (*p* < 0.05) or clinically important covariates. All significant factors (*p* < 0.05) were retained in the final model. In univariable and multivariable analysis, the dose of intra‐operative fentanyl equivalents was regarded as categorical variable. Sequential landmark analyses were performed to compare the survival time of patients surviving a minimum of >1, >3, and >5 years from surgery in both high and low dose groups. To ensure the baseline characteristics comparability between high‐dose and low‐dose intra‐operative fentanyl equivalents groups, propensity score matching (PSM) analysis was used to select the nearest propensity score for all variables (with calipers set at 0.2 SD of the logit of the PS) across high dose or low dose in a 1:1 ratio. All analyses were performed using SPSS 26.0 (IBM, Armonk, NY).

## 3 RESULTS

### 3.1 Patient characteristics

**FIGURE 1:**
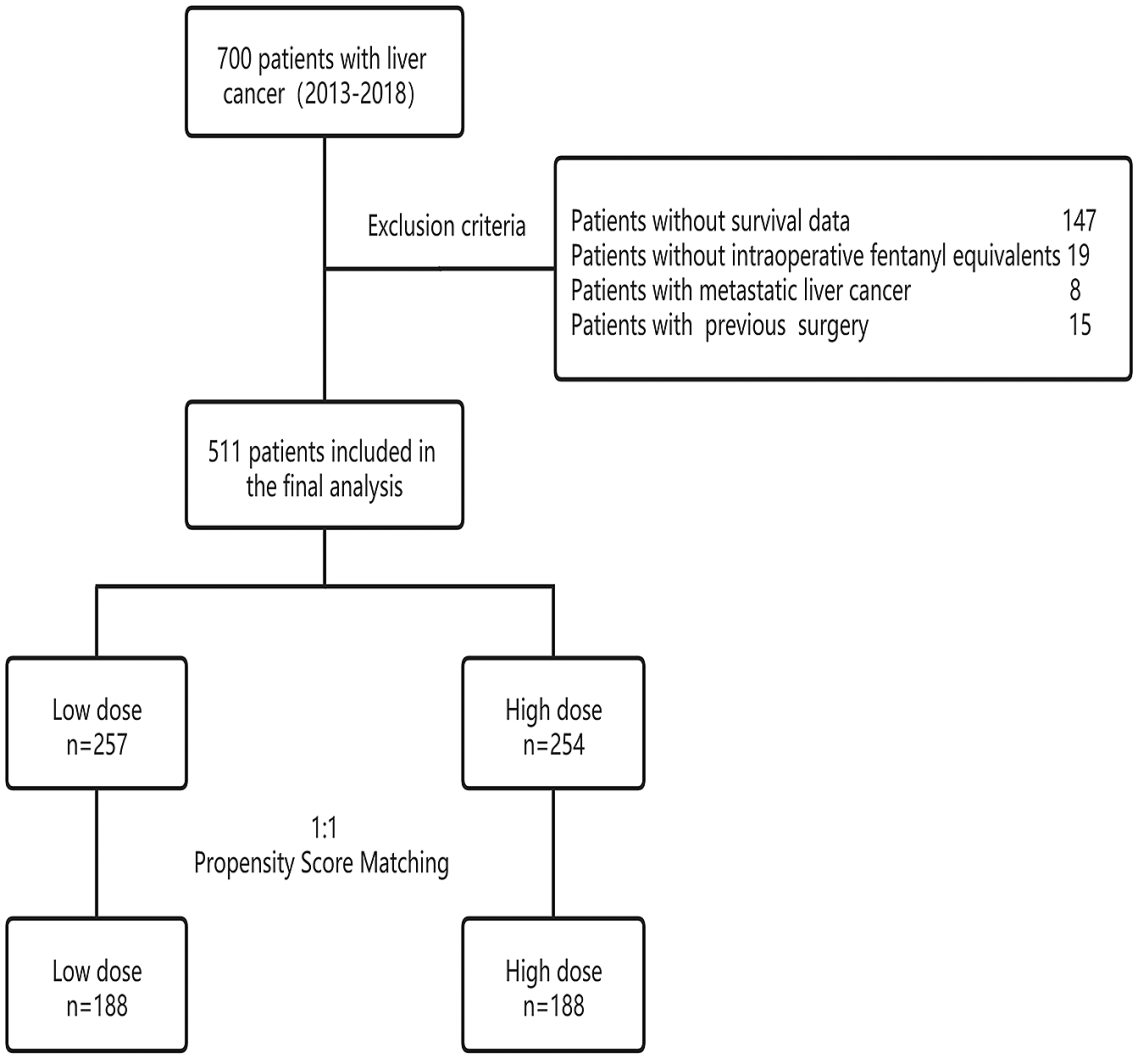
Flow diagram.

Three hundred and seventy six patients were enrolled in the study after selection (Figure 1). Minimum and maximum doses of intra‐operative fentanyl equivalents were 0.154 and 5.112 mg, respectively. The interquartile range of dose of intra‐operative fentanyl equivalents was from 1.100 to 1.986 mg. The median dose of intra‐operative fentanyl equivalents was 1.500 mg and patients were divided into the high‐ and low‐dose groups by this value. Demographic and clinical characteristics of patients were shown in Table 1. There are obvious differences between intra‐operative fentanyl equivalents and BMI (*p* = 0.037), ASA classification (*p* = 0.005), TNM stage (*p* = 0.038), tumor thrombus (*p* = 0.029), blood perfusion (*p* < 0.001) and duration of operation (*p* < 0.001). Age (*p* = 0.363), gender (*p* = 0.321), high blood pressure (*p* = 0.667), diabetes (*p* = 0.902), histopathological classification (*p* = 0.233) and tumor size (*p* = 0.311) did not differ significantly between the groups. After propensity score matching, all variables did not differ significantly between the high and low groups except duration of operation (*p* = 0.001).

**TABLE 1:** Baseline characteristics of patients in both groups

	Overall patients *N* = 511		*p* value	Matched patients *n* = 376		*p* value
Variables	Low‐dose (*n* = 257)	High‐dose (*n* = 254)		Low‐dose (*n* = 188)	High‐dose (*n* = 188)	
Age (year)	54 (26–75)	54 (26–73)	0.363	54 (29–75)	53 (26–73)	0.157
Gender			0.321			0.441
Male	195 (75.9)	202 (79.5)		147 (78.2)	153 (81.4)	
Female	62 (24.1)	52 (20.5)		41 (21.8)	35 (18.6)	
BMI (kg/m^2^)	24.2 (18.1–34.6)	24.8 (18.0–36.2)	0.037	24.2 (18.0–34.6)	24.7 (18.0–36.2)	0.328
High blood pressure			0.667			0.784
Yes	215 (83.7)	216 (85.0)		155 (82.4)	157 (83.5)	
No	42 (16.3)	38 (15.0)		33 (17.6)	31 (16.5)	
Diabetes			0.902			0.719
Yes	21 (8.2)	20 (7.9)		18 (9.6)	16 (8.5)	
No	236 (91.8)	234 (92.1)		170 (90.4)	172 (91.5)	
ASA classification			0.005			0.215
1	7 (2.7)	0		3 (1.6)	0	
2	247 (96.1)	246 (96.9)		182 (96.8)	186 (98.9)	
3	3 (1.2)	8 (3.1)		3 (1.6)	2 (1.1)	
Histopathological classification			0.233			0.492
Hepatocellular carcinoma	246 (95.7)	238 (93.7)		179 (95.2)	176 (93.6)	
Intrahepatic cholangiocarcinoma	10 (3.9)	16 (6.3)		8 (4.3)	12 (6.4)	
Mixed type	1 (0.4)	0		1 (0.5)	0	
TNM stage			0.038			0.165
I	170 (66.1)	145 (57.1)		118 (62.8)	114 (60.6)	
II	49 (19.1)	50 (19.7)		42 (22.3)	33 (17.6)	
III	38 (14.8)	59 (23.2)		28 (14.9)	41 (21.8)	
Tumor size (mm)			0.311			0.757
≤40	138 (53.7)	125 (49.2)		99 (52.7)	96 (51.1)	
>40	119 (46.3)	129 (50.8)		89 (47.3)	92 (48.9)	
Tumor thrombus			0.029			0.410
Yes	34 (13.2)	52 (20.5)		29 (15.4)	35 (18.6)	
No	223 (86.8)	202 (79.5)		159 (84.6)	153 (81.4)	
Blood perfusion			<0.001			0.090
Yes	30 (11.7)	75 (29.5)		30 (16.0)	43 (22.9)	
No	227 (88.3)	179 (70.5)		158 (84.0)	145 (77.1)	
Duration of operation (h)			<0.001			0.001
≤2.170	178 (69.3)	80 (31.5)		110 (58.5)	79 (42.0)	
>2.170	79 (30.7)	174 (68.5)		78 (41.5)	109 (58.0)	

Abbreviations: ASA, American Society of Anesthesiologists physical status; BMI, body mass index; TNM, tumor node metastasis.

### 3.2 Factors associated with RFS after hepatectomy

**FIGURE 2:**
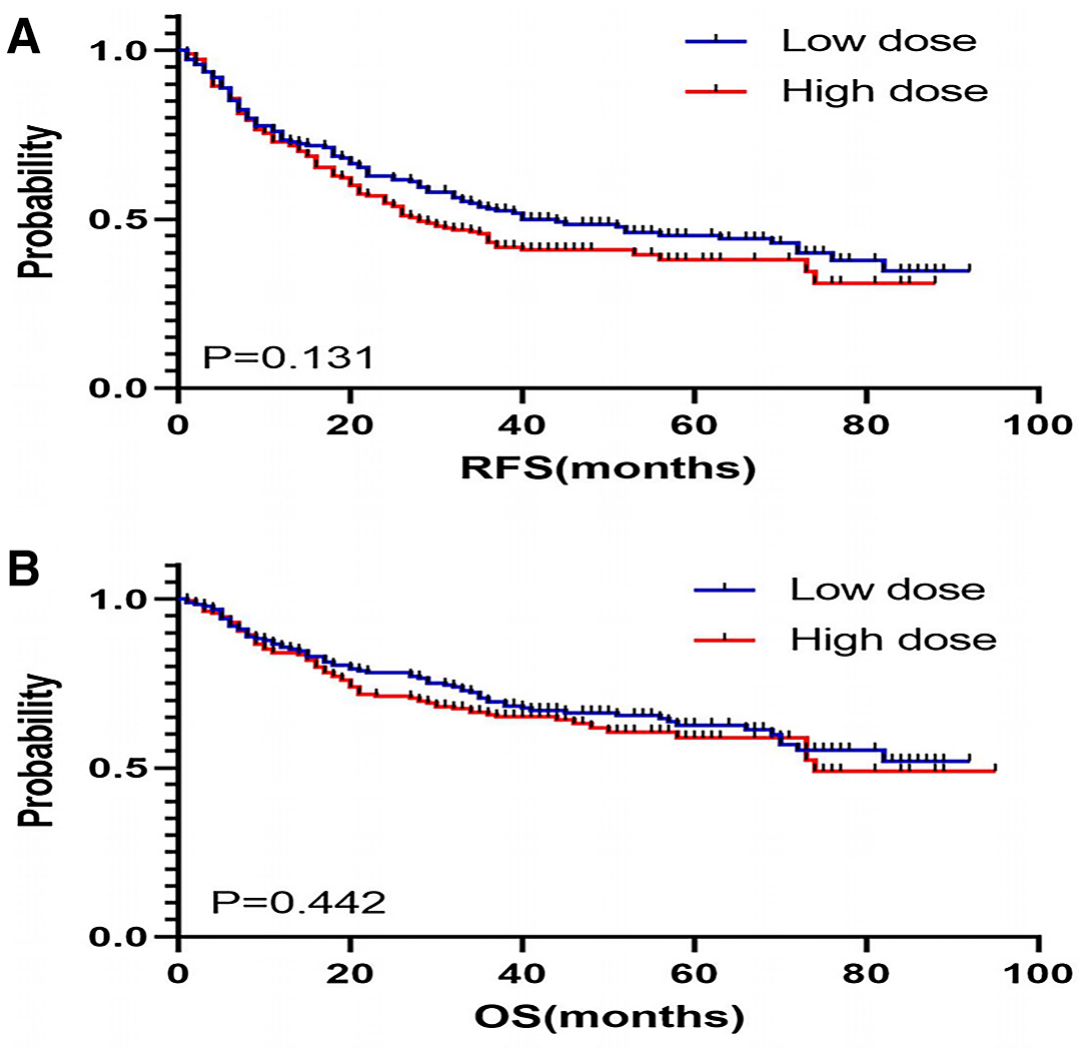
Kaplan–Meier curves for recurrence‐free (A) and overall survival (B) of high‐ and low‐dose groups.

**FIGURE 3:**
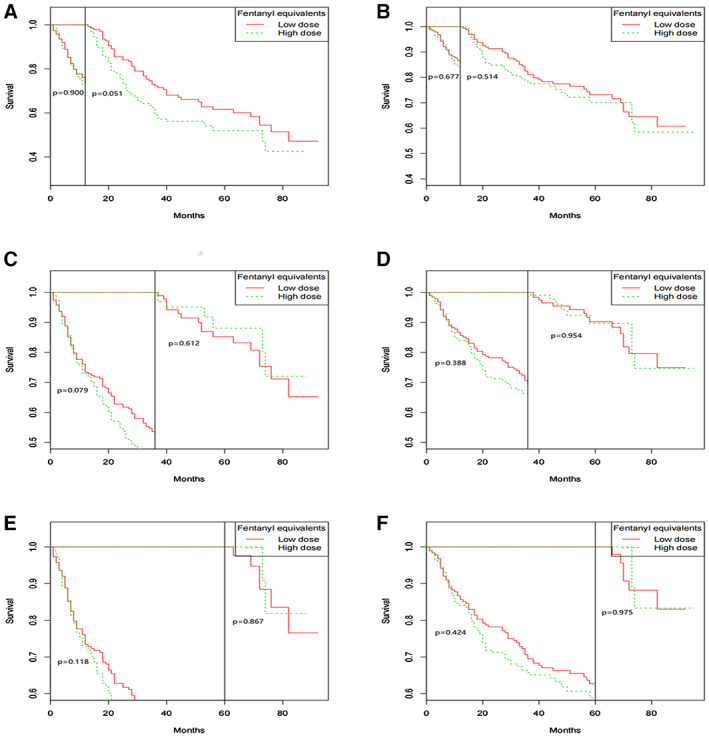
Landmark analyses of recurrence‐free (A‐C) and overall survival (D‐F) for long‐term (>1, >3, and >5 years) survivors.

After propensity score matching, KM curve showed that there was no difference of RFS between high‐ and low‐dose groups (*p* = 0.131) (Figure 2A). The univariable Cox regression analysis found that dose of intra‐operative fentanyl equivalents was not associated with RFS (*p* = 0.136). Histopathological classification (*p* < 0.001), TNM stage (except stage II) (*p* < 0.001), and duration of operation (*p* = 0.005) were risk factors for poor RFS (Table 2). Multivariable analysis showed the dose of intra‐operative fentanyl equivalents was not associated with RFS (HR: 1.119, 95%CI: 0.851 to 1.472, *p* = 0.422) (Table 3). Histopathological classification (hepatocellular carcinoma versus intrahepatic cholangiocarcinoma and mixed, HR, 0.444; 95% CI: 0.274–0.720; *p* = 0.001), TNM stage (III versus I, HR: 1.882, 95% CI: 1.345–2.633, *p* < 0.001) were associated with lower RFS (Table 3). Landmark analyses showed that there was no significant difference in 1‐year (72.9% vs. 73.4%, *p* = 0.900), 3‐year (43.0% vs. 53.0%, *p* = 0.079) and 5‐year RFS (37.9% vs. 45.2%; *p* = 0.118) between high and low groups (Figure 3). The impact of intra‐operative fentanyl equivalents for long‐term survivors of >1, >3, and >5 years are shown in Figure 3. The number of long‐term survivors of >1 year was 138 cases in the low‐dose group and 137 cases in the high‐dose group. The number of long‐term survivors of >3 years was 89 cases in the low‐dose group and 64 cases in the high‐dose group. The number of long‐term survivors of >5 years was 44 cases in the low‐dose group and 18 cases in the high‐dose group. RFS of higher dose fentanyl equivalents group was shorter compared to lower dose fentanyl equivalents group for long‐term survivors of >1 year although no statistical significance was found (*p* = 0.051). However, there was no difference of RFS between higher and lower dose of fentanyl equivalents group for long‐term survivors of >3 (*p* = 0.612), and >5 years (*p* = 0.867).

**TABLE 2:** Univariable analysis of RFS and OS

Variables	RFS		OS	
	HR (95% CI)	*p*‐value	HR (95% CI)	*p*‐value
Age (year)	0.986 (0.971–1.001)	0.064	1.001 (0.983–1.019)	0.938
Male (ref: Female)	1.089 (0.775–1.530)	0.622	0.931 (0.623–1.391)	0.726
BMI (kg/m^2^)	1.034 (0.993–1.078)	0.108	1.017 (0.968–1.070)	0.499
High blood pressure (ref: No)	1.066 (0.745–1.525)	0.725	0.932 (0.605–1.435)	0.749
Diabetes (ref: No)	1.414 (0.925–2.162)	0.110	1.235 (0.724–2.108)	0.439
ASA (ref: 1)				
2	0.526 (0.168–1.647)	0.270	0.674 (0.167–2.727)	0.580
3	0.347 (0.058–2.084)	0.247	0.795 (0.112–5.667)	0.819
Histopathological classification (ref: Intrahepatic cholangiocarcinoma and mixed)				
Hepatocellular carcinoma	0.395 (0.245–0.635)	<0.001	0.223 (0.135–0.370)	<0.001
TNM (ref: I)				
II	1.131 (0.789–1.621)	0.503	1.416 (0.924–2.169)	0.110
III	2.123 (1.540–2.928)	<0.001	2.773 (1.897–4.055)	<0.001
Tumor size > 40 mm (ref: ≤40 mm)	1.287 (0.986–1.679)	0.064	1.194 (0.861–1.655)	0.288
Tumor thrombus (ref: No)	1.067 (0.743–1.532)	0.727	1.504 (0.999–2.264)	0.051
Blood perfusion (ref: No)	1.347 (0.984–1.843)	0.063	1.239 (0.840–1.827)	0.280
Duration of operation, >2.170 h(ref: ≤2.170 h)	1.471 (1.127–1.922)	0.005	1.853 (1.327–2.587)	<0.001
Fentanyl equivalents, >1.500 mg(ref: ≤1.500 mg)	1.225 (0.938–1.601)	0.136	1.136 (0.819–1.576)	0.444

Abbreviations: ASA, American Society of Anesthesiologists; BMI, body mass index; CI, confidence interval; HR, hazard ratio; OS, overall survival; RFS, recurrence‐free survival; TNM, tumor node metastasis.

**TABLE 3:** Multivariable cox proportional of RFS and OS

Variables	RFS		OS	
	HR (95% CI)	*p*‐value	HR (95% CI)	*p*‐value
Histopathological classification (ref: Intrahepatic cholangiocarcinoma and mixed)				
Hepatocellular carcinoma	0.444 (0.274–0.720)	0.001	0.251 (0.150–0.421)	<0.001
TNM (ref: I)				
II	1.094 (0.761–1.573)	0.626	1.303 (0.846–2.005)	0.229
III	1.882 (1.345–2.633)	<0.001	2.247 (1.510–3.346)	<0.001
Duration of operation, >2.170 h(ref: ≤2.170 h)	1.264 (0.954–1.673)	0.102	1.533 (1.079–2.178)	0.017
Fentanyl equivalents, >1.500 mg(ref: ≤1.500 mg)	1.119 (0.851–1.472)	0.422	0.939 (0.668–1.319)	0.715

Abbreviations: CI, confidence interval; HR, hazard ratio; OS, overall survival; RFS, recurrence‐free survival; TNM, tumor node metastasis.

### 3.3 Factors influencing OS after hepatectomy

After propensity score matching, the difference between high‐ or low‐dose groups and OS (*p* = 0.442) could not be observed in the KM curve (Figure 2B). After the univariable Cox regression analysis, we found that dose of intra‐operative fentanyl equivalents was not a risk factor for OS (*p* = 0.444) (Table 2). Histopathological classification (*p* < 0.001), TNM stage (except stage II) (*p* < 0.001) and duration of operation (*p* < 0.001) were associated with lower OS (Table 2). Higher dose of intra‐operative fentanyl equivalents was not an independent predictor for OS in multivariable analysis (HR: 0.939, 95%CI: 0.668–1.319, *p* = 0.715) (Table 3). Histopathological classification (hepatocellular carcinoma versus intrahepatic cholangiocarcinoma and mixed, HR, 0.251; 95% CI: 0.150–0.421; *p* < 0.001), TNM stage (III vs. I, HR: 2.247, 95%CI: 1.510 to 3.346, *p* < 0.001), duration of operation longer than 2.17 h (HR: 1.533, 95%CI: 1.079–2.178, *p* = 0.017) were independent predictors for worse OS after they were included in multivariable analysis (Table 3). We conducted landmark analyses and found that there was no significant difference between patients of high and low dose groups during follow‐up in 1‐year (84.0% vs. 85.6%, *p* = 0.677), 3‐year (65.7% vs. 69.5%, *p* = 0.388) and 5‐year OS (58.9% vs. 62.7%; *p* = 0.424; Figure 3). The impact of intra‐operative fentanyl equivalents for long‐term survivors of >1, > 3, and >5 years are also shown in Figure 3. The number of long‐term survivors of >1 year was 161 cases in the low‐dose group and 158 cases in the high‐dose group. The number of long‐term survivors of >3 years was 119 cases in the low‐dose group and 102 cases in the high‐dose group. The number of long‐term survivors of >5 years was 57 cases in the low‐dose group and 29 cases in the high‐dose group. There was no difference of OS between high‐dose and low‐dose fentanyl equivalents group for long‐term survivors of >1 (*p* = 0.514), >3 (*p* = 0.954), and >5 years (*p* = 0.975).

## 4 DISCUSSION

This is the first study to evaluate the impact of intra‐operative fentanyl equivalents on the recurrence and survival time of primary liver cancer patients after hepatectomy. Results of present study showed no association between the dose of intra‐operative fentanyl equivalents and RFS or OS. This result suggested that cancer recurrence or metastasis of patients of primary liver cancer after curative surgery is not impacted by the intra‐operative consumption of fentanyl equivalents.

There have been increasing studies examined the association between the dose of intra‐operative opioids and survival outcomes after cancer surgery. Retrospective studies reported that higher dose of intra‐operative opioids was associated with worse survival in cancer patients, such as stage I non‐small cell lung cancer,^7^ prostate cancer,^8^ renal cancer,^9^ laryngeal cancer,^10^ oral cancer,^11^ and esophageal squamous cell carcinoma.^12^ On the contrary, several studies found no correlation between the dose of intra‐operative opioids and prognosis such as in breast cancer,^13^ esophageal adenocarcinoma,^12^ and colorectal cancer.^14^ Until now, there has been no definite conclusion whether the amounts of intra‐operative opioids may impact the survival of cancer patients or not.

Opioids may impact the survival of cancer patients through several mechanisms: Direct effect on tumor cell growth, on angiogenesis, and modulation of immune function. Experimental studies suggested that opioids can stimulate the angiogenesis and promote metastasis of the tumor by binding with mu‐opioid receptor.^15^ However, the effects of opioids on tumor growth are mostly based on chronic use of opioids instead of acute intra‐operative use. It is questionable to associate the survival time to the dose of intra‐operative opioids. On the other hand, opioid analgesics used in the perioperative period may contribute to the future metastasis through suppression of antitumoral cellular immunity during the operation.^6^, ^16^, ^17^, ^18^, ^19^, ^20^, ^21^, ^22^, ^23^

The strength of this study is that we collected comprehensive pre‐operative clinical information. Because there were differences in variables between high‐dose and low‐dose intra‐operative fentanyl equivalents groups, we used propensity score match to mimic randomized controlled study by minimizing the influence of selection bias and confounding factors between two groups. After PSM, the baselines of patients in two groups were comparable. Univariable and multivariable analysis was applied to after PSM to explore the potential risk factors which many impact RFS or OS. Our study showed that TNM stage and clinicopathological type were associated with both RFS and OS in both univariable and multivariable analysis. The above association is in line with the clinical situation of real world. Secondly, we applied landmark analysis to explore the impact of dose of intra‐operative fentanyl equivalents on long‐term survivors of >1, >3, and >5 years. Landmark analysis design time points in the follow‐up period and only analyze only those subjects who have survived until the landmark time.^24^

Our study had limitations. This was a retrospective study, we have scant information of preoperative and post‐operative anti‐tumor treatments which may influence the outcome after surgery. Similarly, we have little information about the dose of opioids used postoperatively for pain control. Further studies such as randomized clinical trials are needed, prospective well‐designed and multicenter studies can be required too.

In conclusion, this is the first study that investigates the association between intra‐operative fentanyl equivalents and survival outcomes in primary liver cancer patients. The dose of intra‐operative fentanyl equivalents had no impact on RFS and OS after surgery in patients with primary liver cancer.

## AUTHOR CONTRIBUTIONS

Liuyuan Zhao and Lei Teng: Both authors contributed equally to this work, study design and implementation; Wenhui Zhang, Shiyan Lin, and Xuejiao Liu: data collection; Junzhu Dai, Hongxue Shao, Xiaoshi Li, and Quan Liu: result analysis; Huichao Zou: responsible for the overall content of the manuscript.

## FUNDING INFORMATION

The work was supported by Nn10 Research Award to Dr. Huichao Zou from Harbin Medical University Cancer Hospital.

## CONFLICT OF INTEREST

The authors disclose no potential conflicts of interest.

## ETHICS STATEMENT

This study was approved by the Institutional Review Board of Harbin Medical University Cancer Hospital (2020‐412R). Informed consent from the patients was waived because the nature of this retrospective study was reanalyzing of existing data which does not involve any potential risks and benefits to the patients.

## Data Availability

The data that support the findings of this study are available on request from the corresponding author. The data are not publicly available due to privacy and ethical restrictions.
